# Fracture Resistance and Wear Behavior of Ultra‐Thin Occlusal Veneers Made From Translucent Zirconia Ceramics Bonded to Different Tooth Substrates

**DOI:** 10.1111/jerd.13409

**Published:** 2025-01-07

**Authors:** Nele Fulde, Sebastian Wille, Matthias Kern

**Affiliations:** ^1^ Department of Prosthodontics, Propaedeutics and Dental Materials, School of Dentistry Christian‐Albrechts University at Kiel Kiel Germany

**Keywords:** all‐ceramic, fracture resistance, occlusal veneers, wear behavior, zirconia

## Abstract

**Objective:**

Investigation of the mechanical properties of occlusal veneers made from zirconia with varying translucency, bonded to different tooth substrates.

**Materials and Methods:**

Sixty‐four extracted molars were divided into two groups: preparation within enamel (E) or extending into dentin (D). Veneers were milled from four zirconia ceramics (*n* = 8): 5Y‐TZP (HT), a multilayer of 5 and 3Y‐TZP (GT), 3Y‐TZP (LT), and 4Y‐TZP (MT). After bonding, specimens underwent thermocycling (7500 cycles; 5°C–55°C) and chewing simulation (1,200,000 cycles; 10 kg), followed by investigation of the wear behavior using optical and laser scanning microscopy. Specimens were then loaded to examine fracture resistance.

**Results:**

All specimens except one in the HT group survived artificial aging. Zirconia wear was below the resolution of the 3D scanner, with no significant difference in antagonist vertical loss across materials (*p* > 0.05). Fracture resistance was significantly higher in E‐LT compared with E‐HT (*p* = 0.003). No significant differences (*p* > 0.05) were observed in fracture resistance between materials bonded to dentin, or between the materials within different substrates.

**Conclusions:**

Veneers made of 3 and 4Y‐TZP zirconia showed promising in vitro performance and fracture resistance, regardless of the tooth substrate. All zirconia ceramics exhibited favorable wear behavior.

**Clinical Significance:**

As tooth wear rates increase, the use of minimally invasive occlusal veneers is becoming more important. The goal is to create restorations that are both esthetically pleasing and mechanically durable, while keeping them thin to minimize occlusal preparation and still ensure sufficient fracture resistance. Ultra‐thin (0.3 mm in the fissures/0.6 mm at the cusps) occlusal veneers made of 4Y‐TZP, multilayer and 3Y‐TZP zirconia ceramics survived chewing simulation corresponding 5‐years. All zirconia restorations showed wear‐resistant behavior.

## 1 Introduction

As the abrasion and erosion rate of dental hard tissues is increasing, especially in developed countries, the restoration of occlusal surfaces with minimally invasive occlusal veneers is becoming increasingly important [1]. Restorations should be esthetically pleasing but also withstanding intraoral loading conditions. Likewise, the restorations should be as thin as possible in order to reduce the required occlusal preparation to a minimum, but still have sufficient fracture resistance as fractures are the most frequent failures of all‐ceramic restorations [2]. Factors influencing the fracture strength of occlusal veneers are identified in the literature as the preparation design, the thickness of the restoration, the luting technique and the bonding surface [3, 4]. The manufacturer specifies 1 mm as the minimum occlusal layer thickness for adhesively bonded IPS e.max occlusal veneers [5].

The use of adhesively bonded lithium disilicate ceramics for occlusal veneers is already established and approved, but zirconia with its higher biaxial flexural strength and fracture resistance may be an alternative [6]. The fracture resistance of a dental zirconia depend on its crystal structure [7]. By incorporating yttrium oxide (Y_2_O_3_) into the zirconium dioxide (ZrO_2_) crystal lattice, the tetragonal or cubic phase is partially stabilized at room temperature and the transformation into the monoclinic phase is suppressed, preventing volume expansion and residual stresses [8]. According to the amount of Y_2_O_3_ (mol%) used, 3Y‐TZP, 4Y‐TZP, and 5Y‐TZP zirconia ceramics exist on the market with respectively increasing proportion of cubic and decreasing proportion of tetragonal phases [9]. A high proportion of cubic phase results in high translucency and therefore enhanced esthetics, but lower strength due to the reduced transformation toughening (transformation from tetragonal to monoclinic phase). Occlusal veneers made of 3Y‐TZP zirconia with a thickness of 0.5 and 1 mm exhibit significantly higher fracture resistance compared with veneers made of lithium disilicate [10, 11, 12]. Similar studies comparing the survival rate and fracture resistance of occlusal veneers made of 3Y‐, 4Y‐, and 5Y‐TZP zirconia have not been published to date. The mechanical properties of occlusal veneers made of multilayer zirconia have also not yet been examined. An evaluation of the fracture resistance of translucent zirconia veneers with improved esthetic properties therefore appears to be useful.

Adhesive bonding supports the tooth structures and has a great impact on the longevity of ceramic restorations of minimally invasive, non‐retentive preparations [13]. The bond strength between zirconia and tooth structure is influenced by the luting material and varies depending on bonding to enamel or dentin [14]. The adhesive bond of a self‐etching 1‐component adhesive system to the tooth structure has the advantage over multi‐bottle systems of simplicity and improved dentin bonding. An additional selective enamel etching significantly increases the bond strength to enamel [15].

The wear behavior of dental restorations should correspond as closely as possible to the physiological abrasion of the enamel of 20–40 μm per year in order to avoid disturbances in the occlusion or premature loss of the restoration [16]. Zirconia blocks tested under chewing simulation showed no significant differences between 3Y‐, 4Y‐, and 5Y‐TZP, neither in their own substance loss nor in their antagonist wear [17]. Similar studies are lacking for extracted human teeth embedded in artificial periodontium. Very well polished zirconia ceramics are considered to be low‐wear and show less material loss and wear of the antagonist than lithium disilicate ceramics [17]. The substance loss of the antagonist by zirconia ceramics is less dependent on the hardness of the material than on its superficial roughness [18]. A multi‐step polishing of the zirconia has a positive effect on the surface structure and thus a lower friction towards the antagonist [19].

The aim of the present laboratory study was to investigate the mechanical behavior of thin occlusal veneers made of zirconia, which were monolithically milled from different materials and adhesively bonded to different tooth substrates. Survival rate after chewing simulation corresponding to 5 years in vivo, fracture resistance and wear behavior were examined. The first hypothesis of this study was that neither the bonding tooth substrate nor the type of zirconia will influence the fracture resistance of occlusal veneers. The second hypothesis stated that the type of zirconia also had no influence on the wear behavior of the occlusal veneers.

## 2 Materials and Methods

Extracted human third molars (*n* = 64) without caries or fillings were collected after informed consent in accordance with the local ethic committee (Ethic votum: Kiel University D528/19) and cleaned of tissue debris. The teeth selected for this study were chosen to ensure they were as uniform in size and shape as possible. For this reason, only maxillary molars that did not exceed or were below a mesiodistal width of 8–10.5 mm and a bucco‐lingual length of 9–12 mm were used.

The molars were preserved in a 0.1% thymol solution for 2 weeks. To avoid any influence on the bond strength, the teeth were subsequently stored in isotonic NaCl solution at 4°C [20]. When starting the experiments they were embedded in standard brass cylinders: After teeth cleaning with a scaler, they were positioned along their longitudinal axis within a temporary cylinder and the space between the tooth root and the cylinder was filled with wax (Surgident Periphery wax, Kulzer, Wehrheim, Germany) up to 2 mm apical to the cemento‐enamel junction (CEJ). This position within the cylinder was held in place by taking impressions using doubling compound (Dublisil 15, Dreve Dentamid, Unna, Germany) before the temporary cylinders and wax were removed. As a result, the roots were exposed up to 2 mm apical to the CEJ. This was followed by drilling in a mesiodistal direction through the apical third of the roots, into which a thin steel bar (0.9 mm) could be inserted to prevent rotation. A simulation of the periodontal ligament was achieved by coating the roots with a gum resin (Anti‐Rutsch‐Lack, Wenko‐Wenselaar, Hilden, Germany) in a thickness of 0.2 mm. A uniform thin coating was ensured by dipping once and removing the excess apically. After placing the final metal cylinder (diameter: 15 mm) in the impression of the impression material, these were filled with autopolymerizing acrylic resin (Technovit 4000, Kulzer, Wehrheim, Germany) to simulate the bone.

**FIGURE 1:**
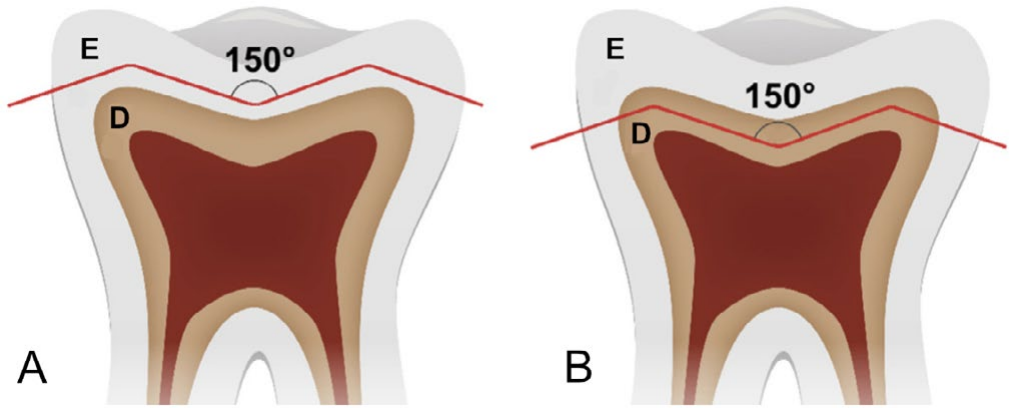
Preparation design: (A) preparation within enamel (Group E) and (B) within dentin with enamel margins (Group D) (D = dentin, E = enamel) [21].

The embedded molars were divided into two groups (*n* = 32 each). An anatoform, non‐retentive preparation of the teeth was performed under water cooling using medium‐grit cylindrical diamond burs, which intended to simulate defects due to abrasion and erosion. This extended within the enamel (E) for one group and into the dentin with margins in enamel (D) for the other. The angle between the cusps was 150° in bucco‐lingual and mesiodistal direction to standardize the preparation geometry (Figure 1), which was checked using a template. To ensure consistency, all preparations were conducted by a single individual with expertise in the procedure.

**FIGURE 2:**
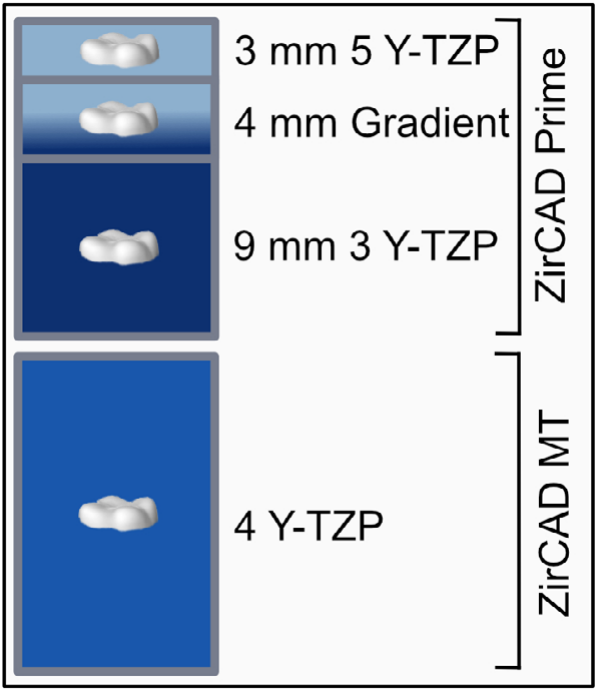
Distribution of the layers of the zirconia discs.

**FIGURE 3:**
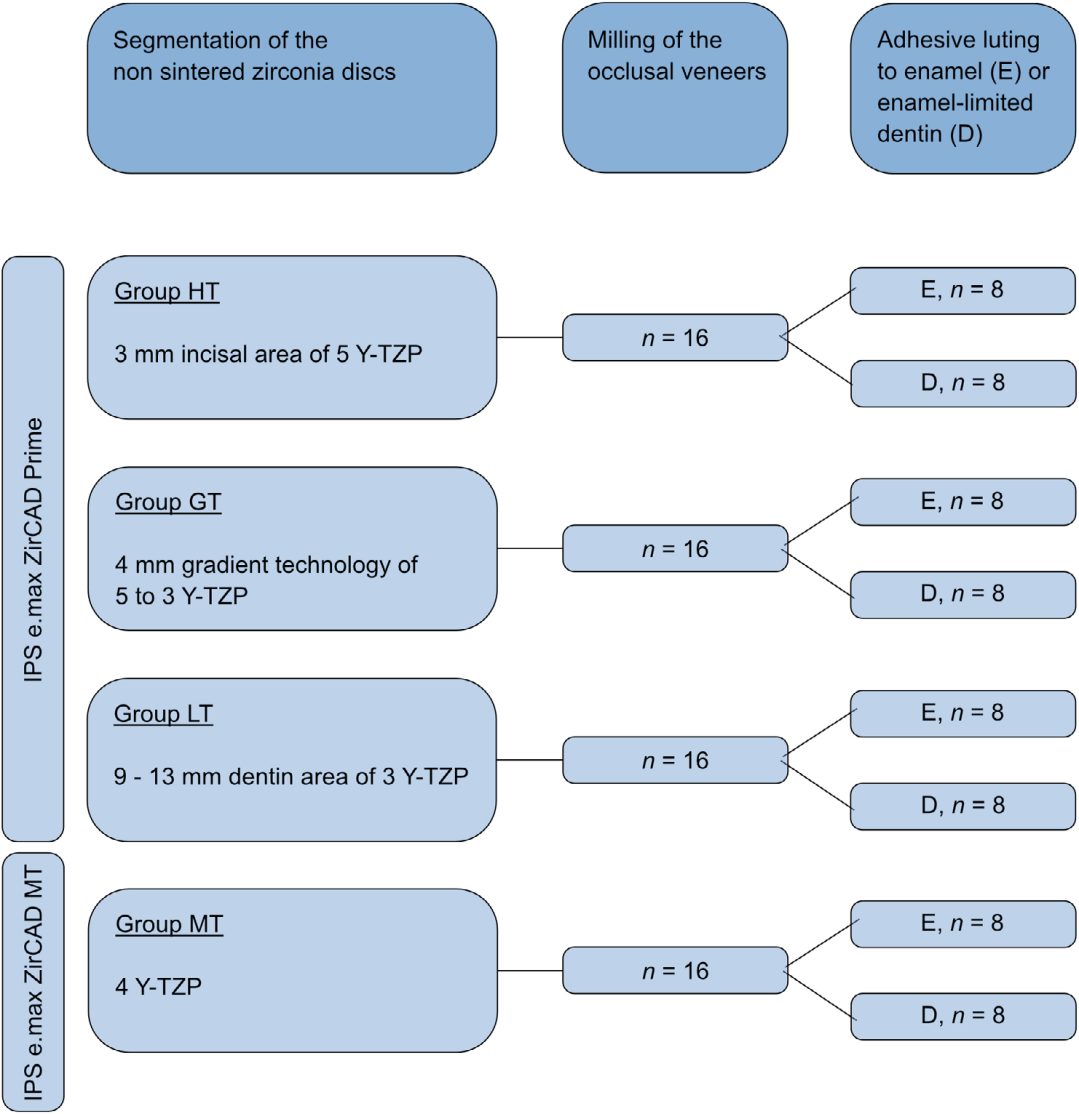
Flow chart of the study design.

After preparation of the occlusal surface, the occlusal veneers were fabricated using CAD/CAM technology: The scan of the teeth (D 900 3D scanner, 3 Shape A/S, Copenhagen, Denmark) was followed by the semi‐anatomical design of the veneers (3 Shape Dental System Premium, 3Shape A/S, Copenhagen, Denmark), which served a standard layer thickness of 0.3 mm in the fissures and 0.6 mm at the cusps. The virtual data were transferred to the milling program (Zenotec CAM V3, Wieland Dental, Pforzheim, Germany) in STL format. The distribution of the layers was obtained from the manufacturer (graphically represented in Figure 2). The virtual veneers were positioned in the zirconia discs according to the study design (Figure 3): In the IPS e.max ZirCAD Prime Disc (Ivoclar, Schaan, Liechtenstein) in three different layers. Within the upper 3 mm, corresponding to the translucent incisal zone of 5Y‐TZP (group HT). In the middle 4 mm, which represents a transition zone from 3Y‐TZP to 5Y‐TZP at a fixed and thus comparable height (group GT). And finally, within the lower 9 mm, corresponding to the less translucent dentin zone of 3Y‐TZP (group LT). The fourth group was positioned in the uniform IPS e.max ZirCAD MT Disc (Ivoclar, Schaan, Liechtenstein) made of 4Y‐TZP (group MT).

The veneers were milled from the above‐mentioned unsintered zirconia ceramics (Zenotec Select Hybrid, Wieland Dental, Pforzheim, Germany) and sintered (Controltherm P310, Nabertherm, Lilienthal, Germany). Milling and Sintering were carried out according to the manufacturer's instructions. Finally, a step‐by‐step polishing of the restorations was performed using oxide ceramic polishers (light blue and dark blue flame, OptraFine, Ivoclar, Schaan, Liechtenstein), clamped in a reducing contra‐angle handpiece under water cooling, at a maximum of 15,000 rpm and 2 N pressure force.

The cleaned ceramic bonding surfaces were colored with waterproof blue felt‐tip pen (Staedtler Lumocolor permanent, strength M) and these surfaces were then air‐abraded with alumina particles (50 μm Al_2_O_3_ at 1 bar) until the blue color had been completely removed. The ceramic restorations were placed in an ultrasonic bath with 99% isopropanol for 3 min each, dried, and an MDP‐containing universal primer (Monobond Plus, Ivoclar, Schaan, Liechtenstein) was applied for 60 s and blown with oil‐free air for 5 s.

Surface treatment of the prepared tooth surfaces followed. A phosphoric acid gel (37%) was applied to the enamel for 15 s using the selective etching technique. In accordance with the study design, the etching of the enamel groups (E) was carried out over the entire area of preparation, while in the group with dentin and enamel margins (D) only the enamel margins were etched. The phosphoric acid was sprayed off for 30 s and teeth were dried. The tooth surfaces were rubbed with a self‐etching tooth primer (Adhese Universal, Ivoclar, Schaan, Liechtenstein) for 20 s, then air‐dried and light‐cured for 10 s (Radii‐Cal LED curing lamp, SDI Limited, Bayswater Victoria, Australia). A dual‐curing luting composite (Variolink Esthetic DC, Ivoclar, Liechtenstein) was applied from the auto mix syringe to the pretreated restorations, which were placed on the tooth surfaces and excess cement removed. The restoration margins were covered with a glycerin gel (Liquid Strip, Ivoclar, Schaan, Liechtenstein) to prevent an oxygen inhibition layer. This was followed by light polymerization for 10 s per side and from occlusal. After spraying off the glycerin gel, post‐curing took place in a water bath at 37°C for 3 days. Finally, all specimens were scanned using a 3D scanner (D 900 3D Scanner, 3 Shape A/S, Copenhagen, Denmark).

**FIGURE 4:**
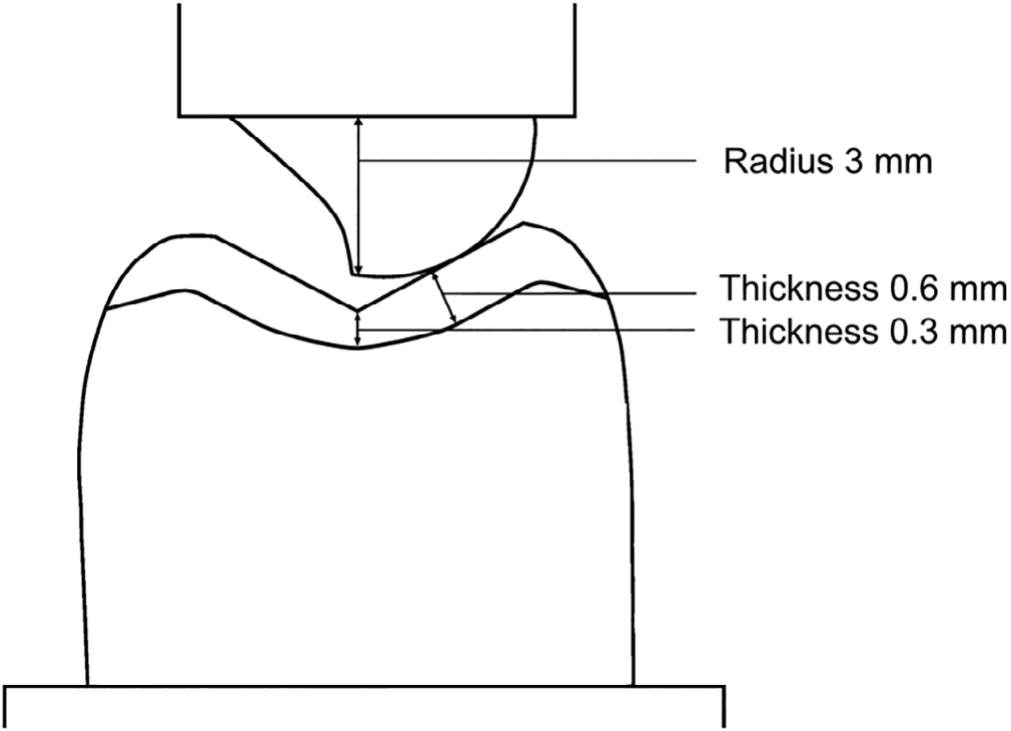
Illustration of the specimen and the steatite ceramic ball antagonist during chewing simulation. The steatite ceramic ball was positioned so that it first hit the supporting cusp slope 2 mm away from the central fissure and then slid down 0.3 mm towards the central fissure.

Thermocycling (Thermocycling Apparatus, Willytec, Munich, Germany) was performed for 7500 cycles at a thermocycling of 5°C and 55°C with a respective dwell time of 30 s in tap water. Dynamic loading was then carried out in a chewing simulator (chewing simulator CS4, SD Mechatronik, Feldkirchen‐Westerhan, Germany). This chewing simulation was performed for 1,200,000 cycles under a load of 98 N at a frequency of 2.4 Hz with additional integrated thermocycling for 4500 cycles of 5°C and 55°C for 30 s each. A descent speed of 30 mm/s, ascent speed of 55 m/s, and a vertical movement of 6 mm were selected. To simulate the antagonist, a customized steatite ceramic ball (Ø 6 mm) (Hoechst Ceram Tec, Wunsiedel, Germany) was used (Figure 4). The initial contact point of the steatite ceramic ball was marked and checked using occlusion foil and selected so that it first hit the supporting cusp slope 2 mm away from the central fissure, where the buccal fissure meets the central fissure, and then slid down 0.3 mm towards the central fissure (but did not reach it).

To evaluate the survival rates, the artificially aged specimens were examined for damage using a stereo microscope (Wild M420, Heerbrugg, Switzerland). In addition, images of veneer surfaces were documented using photographs (Leica DC 100, Leica Microsystems, Cambridge, UK). Specimens that showed a visible chipping under the microscope were classified as a failure. A partial failure occurred when the specimens showed macroscopic or microscopic cracks, which, however, did not affect the integrity of the veneer or the adhesive bond.

Those specimens that survived the dynamic loading without damage were then statically loaded in a universal testing machine (Zwick Z010/TN2A, Ulm, Germany) until fracture. A steel ball (Ø 6 mm) was positioned centrally on the central fissure and the load was applied by means of a compression at a crosshead speed of 2 mm/min (1‐step unconfined compression test). To obtain a homogeneous load on the triangular beads, a 0.6 mm thick tin foil was placed between the occlusal restoration and the steel ball. A computer software (testXpert II, Zwick, Ulm, Germany) recorded the maximum load‐bearing capacity up to fracture formation (N) of the specimens with a resolution of 2 mV/V. The occurrence of the fracture was monitored by simultaneous video recording.

**FIGURE 5:**
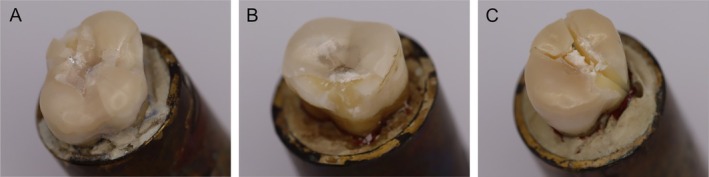
Failure modes after quasi static loading. (A) Cohesive fracture within the restoration without involving the tooth structure (intact tooth) (I); (B) fracture within ceramic and tooth structures (II); (C) longitudinal fracture of the restoration and tooth involving the root (catastrophic fracture) (III).

The evaluation of the failure mode followed. In order to evaluate the failure mode, the specimens were analyzed under an LED light source and a stereomicroscope (Wild M420) at 20× magnification. Three fracture modes were distinguished (Figure 5):

- Cohesive fracture within the restoration without involving the tooth structure (intact tooth).
- Fracture within ceramic and tooth structures.
- Longitudinal fracture of the restoration and tooth involving the root (catastrophic fracture).

Investigation of the wear behavior of the unfractured specimens and the steatite ceramic balls followed after dynamic and cyclic loading. The zirconia restorations were again scanned using a 3D scanner as described above. The measurement inaccuracy of the 3D scanner according to the manufacturer was approximately 10 μm. The scans before and after loading were compared by superimposing them in the Geomagic Wrap software (3D Systems, Moerfelden‐Waldorf, Germany). The vertical loss as well as the volume loss was measured. The steatite spheres, on the other hand, were photographed in semi‐profile with visible extent of the loss. The vertical loss was recorded with Photoshop CS software (version 11.0, Adobe, San Jose, USA) and the volume loss was calculated with the mathematical formula of the sphere segment. As the areas of wear were approximately circular, an average value from a vertical and a horizontal diameter measurement was selected for the diameter for this formula. For the qualitative examination and documentation of the wear behavior of the restorations and the steatite spheres by means of a scanning electron microscope SEM (JSM‐IT200, Jeol, Freising, Germany), precision impressions were taken of the restorations (Express 2Ultra‐Light Body Quick, 3M, Neuss, Germany) and these were poured with epoxy resin (STYCAST 1266, Emerson & Cuming, Westerlo, Belgium). For each group, three of these replicates were selected, which received a 10 nm thick gold coating (Leica EM QSG 100, Wetzlar, Germany) and were examined by means of the SEM.

In order to be able to quantify a change in the surface condition of the zirconia restorations, one replication per group was randomly selected, in which the loaded and unloaded surfaces were compared using a laser scanning microscope (vk‐x 100, Keyence, Osaka, Japan) and the differences could be quantified by measuring the surface roughness.

First, the normal distribution of the maximum fracture resistance data was checked using the Shapiro–Wilk test, revealing that there was no normal distribution of the data. Then, Kruskal–Wallis test was performed and revealed that significant differences existed overall (*p* = 0.017). The groups were then tested in pairs using the Mann–Whitney test and a Bonferroni–Holm correction for multiple comparisons was performed.

A normal distribution of the antagonist wear data was not refuted by the Shapiro–Wilk test. Therefore, the data were statistically analyzed using 1‐way ANOVA followed by Tukey's post hoc test.

All analyses were performed at a significance level of *α* = 0.05.

## 3 Results

**FIGURE 6:**
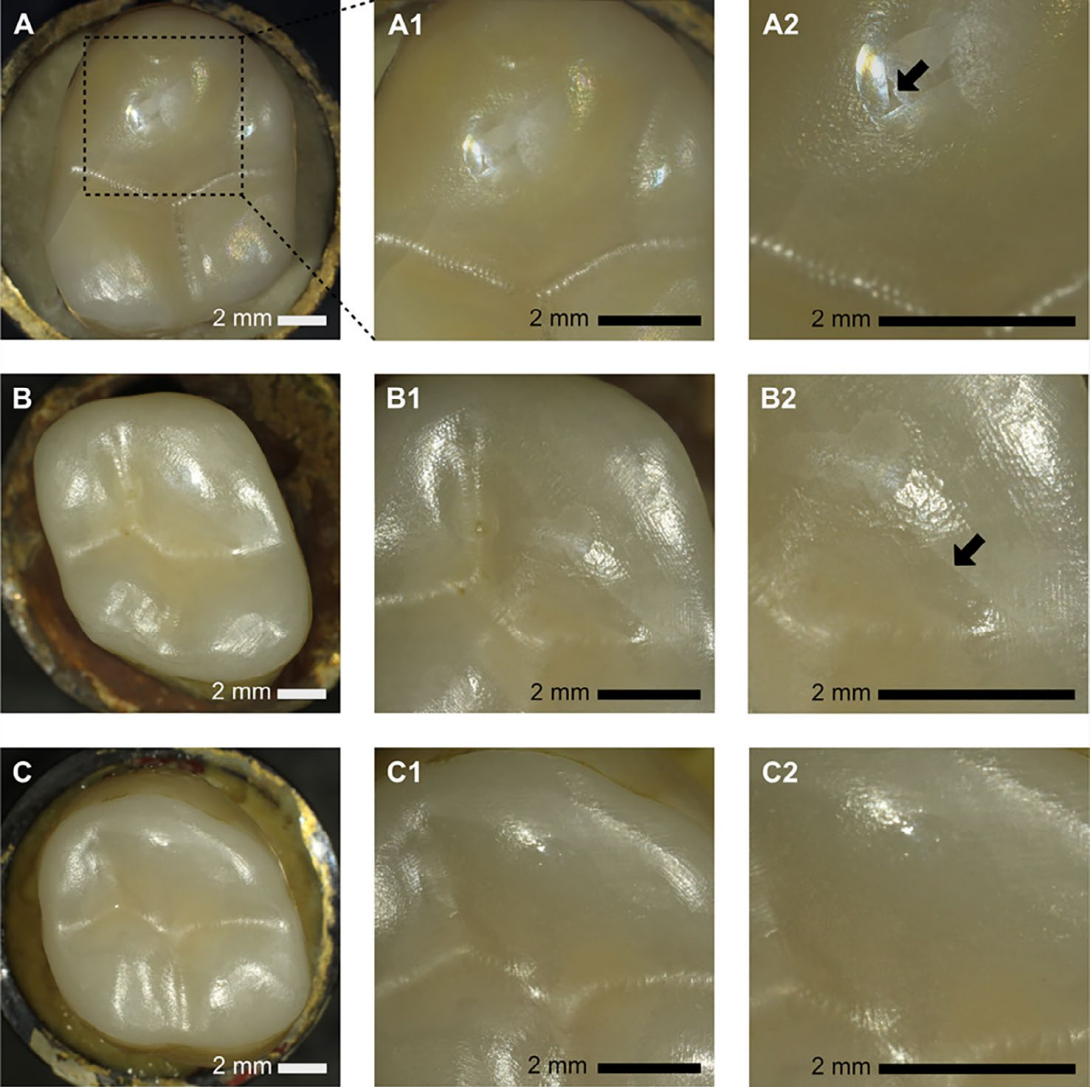
Light microscope images of the occlusal veneers after dynamic loading of 1,200,000 cycles at 98 N. (A) Failure of the zirconia veneer (arrow in A2 shows where chipping occurred). (B) Partial failure of the zirconia veneer (arrow in B2 points to the crack). (C) Complete success of the zirconia veneer.

Overall, 98% of the specimens survived dynamic and cyclic loading. Failure, partial failure and full success could be identified by stereo microscope after dynamic and cyclic loading. One specimen from the group of highly translucent (5Y‐TZP) ceramics (D‐HT) bonded to dentin with margins in enamel showed chipping visible under the microscope, which was rated as a failure (Figure 6A). Partial failures (Figure 6B) affected 7.8% of all specimens, whereby they occurred exclusively in the group of highly translucent zirconia ceramics (5 Y‐TZP). 12.5% of group E‐HT showed partial failure as well as 50% of group D‐HT. Since there was a failure after dynamic loading in one specimen of the D‐HT group, a fracture resistance of 98 N was assigned here. The most frequently identified failure mode after quasi‐static loading was Class II, namely, the chipping of the ceramic including tooth structure for all groups except D‐LT. The most common failure mode identified for these 3Y‐TZP ceramics bonded to dentin and the second most common for these ceramics bonded to enamel was Class III, longitudinal fracture of the entire tooth. The fracture resistance determined for *F*_max_ and the failure modes evaluated follow in Tables 1 and 2.

**TABLE 1:** Fracture resistance (*F*_max_) (N) of the groups.

Group	Bonding surface					
	E			D		
	*M* ± SD	Median	Min	*M* ± SD	Median	Min
			Max			Max
HT	2311 ± 343	2268^A^_a_	1980 3040	2077 ± 851	2440^A^_a_	98 2600
GT	2696 ± 554	2765^A^_a,b_	1643 3584	2213 ± 952	2290^A^_a_	986 3397
LT	3158 ± 457	3255^A^_b_	2280 3790	2642 ± 529	2508^A^_a_	1900 3260
MT	2506 ± 436	2532^A^_a,b_	1731 3144	2078 ± 541	2078^A^_a_	1430 2880

*Note*: Means (*M*) and standard deviations (SD), medians, minima (Min), and maxima (Max). Medians with the same upper case superscript letter within the same row are not statistically different (*p* > 0.05). Medians with the same lower case subscript letter within the same column are not statistically different (*p* > 0.05).

**TABLE 2:** Distribution of the failure modes after quasi static loading of the specimens.

Group	E			D		
	Failure mode			Failure mode		
	I (%)	II (%)	III (%)	I (%)	II (%)	III (%)
HT	12.5	87.5	0	12.5	75	12.5
GT	25	50	25	25	75	0
LT	12.5	50	37.5	37.5	12.5	50
MT	0	100	0	37.5	62.5	0

*Note*: (I) Cohesive fracture within the restoration without involving the tooth structure (intact tooth); (II) fracture within ceramic and tooth structures; (III) longitudinal fracture of the restoration and tooth involving the root (catastrophic fracture).

Considering the type of CAD/CAM material, there was a significant difference in fracture resistance within the enamel bonded restorations, with the LT group showing significantly higher fracture resistance than the HT group (*p* = 0.003). Among the restorations bonded to dentin with margins in enamel, no significant differences were evident with regard to the use of different materials. The statistical analysis of the fracture resistance with regard to adhesive bonding on different substrates did not reveal any statistically significant differences within the material groups.

A qualitative and quantitative examination of the wear of the zirconia restorations and the antagonists was carried out after cyclic and dynamic loading. Based on light and scanning electron microscopic examination of the restorations, there was no visible loss of substance in the area of loading. A quantification of the surface roughness can be seen in Table 3. The quantitative examination of the wear of the ceramics showed such a minimal loss of substance for all specimens that it was within the range of the measurement accuracy of the 3D scanner.

**TABLE 3:** Surface roughness of a randomly selected specimen per group.

Group	Unloaded surface		Loaded surface	
	*R*_*a*_ (μm)	*R*_*z*_ (μm)	*R*_*a*_ (μm)	*R*_*z*_ (μm)
HT	0.315	1.495	0.078	0.342
GT	0.442	2.757	0.213	0.899
LT	0.419	2.086	0.149	0.575
MT	0.498	2.643	0.086	0.361

*Note: R*_*a*_ (arithmetic mean deviation of the assessed profile). *R*_*z*_ (10 point mean roughness).

Medians, minima, maxima, mean values and standard deviations of the vertical substance loss and the volume loss of the steatite ceramic antagonists are given in Table 4. The statistical analysis showed that there was no significant difference in the vertical loss of the antagonists depending on the zirconia material used (*p* > 0.05).

**TABLE 4:** Vertical and volume losses of steatite ceramic balls against zirconia restorations.

Group	Vertical loss (μm)			Volume loss (mm^3^)		
	*M* ± SD	Median	Min	*M* ± SD	Median	Min
			Max			Max
HT	211 ± 80	199	99 400	0.461 ± 0.359	0.366	0.092 1.444
GT	188 ± 61	190	857 329	0.356 ± 0.226	0.334	0.068 0.984
LT	180 ± 45	172	121 283	0.315 ± 0.159	0.275	0.135 0.731
MT	181 ± 45	171	131 287	0.318 ± 0.163	0.270	0.158 0.749

*Note*: Mean values (*M*) and standard deviations (SD), medians, minima (Min), and maxima (Max).

## 4 Discussion

Artificial dynamic loading by using a chewing simulator and thermal cycling shows a good agreement with clinically obtained results [22, 23]. Since the present laboratory study was assuming a loading period of 5 years, it was necessary to go through 1.2 million chewing cycles, based on a natural assumption of 250,000 chewing cycles per year [23, 24, 25, 26]. The chewing force of this study was 98 N corresponding to physiological chewing forces, which was also often used in previous laboratory studies [3, 21, 27].

In this study, all specimens except one, which showed a crack after dynamic loading, were subjected to a quasi‐static fracture load test in a universal testing machine to determine the final fracture resistance. As the loading was purely vertical, the fracture resistance determined cannot be transferred to the clinical situation, in which oblique loads can also occur in the context of parafunctions and dynamic occlusion [28, 29]. The direction of loading is, however, particularly relevant for the fracture strength of ceramic materials, as the distribution of tensile and compressive stresses in the ceramic depends on it [30]. If the tensile stress exceeds the resistance of the ceramic, the restoration will fail in the form of cracks, spalling or complete fracture [30].

Considering the results of artificial aging, most specimens showed full success. Only veneers made of the weaker 5Y‐TZP ceramics showed partial failures and also one failure. Whether the visible cracks of the specimens rated as partial failures would have an influence on the long‐term performance has not yet been investigated. In this study, visible cracks did not necessarily correlate with the lowest maximum fracture strengths of the respective group. A similar study with thin occlusal veneers made of lithium disilicate recorded a success rate of 100% after dynamic loading with some partial failures [3]. One study also investigated the fracture resistance of occlusal veneers made of lithium disilicate, which were bonded without additional enamel etching [21]. The success rate was 50% for purely enamel bonding and 100% for bonding to dentin with enamel margins, and there were partial failures. In both studies, loading with integrated thermocycling was carried out over 600,000 cycles instead of 1,200,000 cycles as in the present study. The amount of specimens showing full success of the GT, LT, and MT groups of the present study exceeds the amount of full success of the two previously mentioned studies, although twice as many cycles were run [3, 21]. This may be due to the improved mechanical properties of zirconia compared with lithium disilicate and the possibility of phase transformation of 3Y‐ and 4Y‐TZP zirconia [6, 7, 31].

The hypothesis that the type of ceramic has no influence on the fracture resistance of the occlusal veneers can be partially confirmed. When the veneers were bonded purely to enamel, the fracture resistance of 3Y‐TZP ceramic was significantly higher than that of the 5Y‐TZP ceramic. The restorations made of 5Y‐TZP ceramic have a reduced number of tetragonal grains due to the increase in the yttrium content, which means a loss of the transformation rate of the tetragonal to the monoclinic phase along a crack [6, 32]. Cracks are thus able to propagate more in 5Y‐TZP than in 3 Y‐TZP ceramics. The result corresponds to laboratory studies in which a significantly higher fracture strength was also observed in specimens made from 3Y‐TZP in contrast to those made from 5Y‐TZP ceramics [6, 7, 32, 33]. The results of the multilayer groups (GT) show the highest standard deviations. Such variability underscores the importance of precise production standards for multilayer zirconia to ensure consistent performance in clinical settings as their fracture resistance is primarily dictated by the weaker 5Y‐TZP phase at the occlusal surface [34].

No significant differences in the type of zirconia ceramic were observed between the other ceramic restorations bonded to enamel and all restorations bonded to dentin with margins in enamel. Adhesive bonding might reduce differences between materials, as the bonding to the tooth substrate improves especially the strength of the weaker materials [35, 36].

All specimens that survived the chewing simulation of 1,200,000 cycles under 98 N and thermocycling showed a maximum fracture strength above the expected maximum masticatory forces in the posterior region. Masticatory forces can range from 200 to 540 N, and up to 880 N in patients with bruxism [37]. The high fracture strength corresponds to a similar study in which the maximum load capacity for 0.5 mm thick zirconia veneers made of 3 Y‐TZP ceramic adhesively bonded to dentin had a median of 2493 N [11]. This value was significantly higher than that of the comparable group of lithium disilicate ceramics. Another study showed similar results, as they reported significantly higher fracture resistance for occlusal veneers made of 4Y‐TZP compared with those made of lithium disilicate ceramics under lateral loading [38]. In the present study, the fracture resistances of occlusal veneers made of zirconia did not exceed those reported in the literature for occlusal veneers made of lithium disilicate ceramics. One study showed that 0.3/0.6 mm occlusal veneers bonded to dentin with margins in enamel had a median fracture resistance of 2370 N [21], the mean value by another study was 3391 N [3]. Occlusal veneers made of 5Y‐TZP zirconia failed to demonstrate statistically significant higher fracture resistance compared with those made of lithium disilicate in two other studies [12, 39].

The hypothesis that the type of substrate to which the veneers were adhesively bonded has no influence on fracture resistance has been statistically proven. There were no statistically significant differences between the restorations bonded to pure enamel or dentin with margins in enamel. Hypothetically, the bond strength of various zirconia ceramics to different tooth substrates does not show any relevant difference, which may be due to the use of a universal adhesive. This allows dentin bonding that is comparable to enamel bonding [40, 41, 42, 43, 44]. The different composition zirconia does not seem to have any influence on their bond strength either. One study compared the fracture strength of occlusal veneers made of zirconia ceramics, which were bonded to dentin, with margins in enamel dentin with an intra‐coronal cavity or dentin with a composite filling [39]. The restorations were bonded using a universal adhesive after prior selective enamel etching. The results showed no statistically significant differences on bonding to the different substrates. Another study with occlusal lithium disilicate veneers, however, found statistically significant differences with regard to the substrate [21]. Restorations with a thickness of 0.3–0.6 mm, which were bonded with a universal adhesive without additional selective enamel etching, showed higher values when bonded to dentin with margins in enamel than when bonded to pure enamel. A study, in which an additional selective enamel etching was carried out, confirmed the results [3]. The lower fracture strength on pure enamel indicate an increased dentin bond strength of the universal adhesive, which also corresponds to the results of another study [45]. Additional increased values could be achieved with immediate dentin sealing when using occlusal veneers made of lithium disilicate ceramics [46]. Such data are still lacking for zirconia restorations.

The substance loss of all four different zirconia ceramics caused by wear after 1,200,000 cycles of chewing simulation was found to be so low that it was within the range of the measurement inaccuracy of the 3D scanner, which according to the manufacturer is approximately 10 μm. This result is similar to that of a study in which the loss of 3Y‐, 4Y‐, and 5Y‐TZP zirconia ceramics after 1,200,000 cycles of chewing simulation under a load of 2 kg was approximately 10 μm [17]. The authors also investigated the wear behavior of lithium disilicate ceramics, which showed a significantly greater substance loss of 880 μm under the same test conditions. They qualitatively described the loss of substance of lithium disilicate ceramics as abrasion, fatigue and corrosion wear in contrast to that of zirconia ceramics. In the present study, too, the surfaces of the zirconium oxide ceramics did not show any visible loss of substance, but only a change in their roughness. There were no significant differences between the different zirconia ceramics.

A correlation between the type of zirconia ceramic and the wear of the antagonists was not found, confirming the second hypothesis. The steatite ceramic balls showed similar loss regardless of whether they were antagonized by 5Y‐TZP, the transitional ceramic, 3Y‐TZP or 4Y‐TZP. The median loss of the steatite ball antagonists ranged from 171 to 199 μm in vertical loss and 0.27 to 0.37 mm^3^ in volume loss. With a clinically assumed 250,000 chewing cycles per year and a 5‐year load corresponding to 1,200,000 cycles, this results in an annual substance loss of about 37 μm and 0.06 mm^3^, which is very similar to the loss in natural dentition, which is 20–40 μm loss of enamel per year [47]. The closer the wear behavior of dental restorations to the physiological wear of enamel, the more likely it is that occlusal disturbances or premature restoration loss will be avoided [48, 49] Studies have found that steatite antagonists experience an annual substance loss of approximately 0.03–0.12 mm^3^ after 1.2 million cycles [17, 50]. Similarly, natural teeth opposing polished zirconia discs show a substance loss of 88–177 μm over 240,000 cycles at 50 N with thermocycling [51]. The highest wear occurs in the first 120,000 cycles, with wear decreasing thereafter, yielding values similar to the current study despite methodological differences. The wear results in these studies align with the current study, although differences in methodology exist, such as using steatite balls instead of natural teeth and anatomically shaped zirconia specimens instead of flat or cuboidal ones. Nevertheless, it can be concluded that zirconia veneers can be used for prosthetic rehabilitation with regard to their favorable wear behavior.

As an example, one specimen per group was randomly selected and the surface roughness was determined to generate an indication of the quality of the finish. Polishing plays a vital role in wear investigations, as the wear of antagonists against zirconia depends significantly on surface roughness rather than hardness [18, 52, 53, 54]. Multi‐step polishing smooths the zirconia surface, reducing friction and antagonist material loss compared with lithium disilicate ceramics [18, 52, 55, 56]. In this study, manual polishing was standardized based on manufacturer instructions, achieving *R*_*a*_ values of approximately 0.3–0.5 μm and *R*_*z*_ values of 1.5–2.7 μm. These results align with existing literature on manually polished zirconia specimens, where *R*_*a*_ values range between 0.28 and 1 μm [19, 57]. However, only randomized samples were examined here, so mean values for all specimens were not calculated, limiting a direct comparison.

A limitation of this study is the inherent nature of the in vitro test setup, as it can only partially replicate the complexity of real clinical conditions. Due to the use of extracted human molars, achieving full standardization of the teeth was challenging because of variations in size and age [58]. Additionally, morphological differences among the extracted teeth could have contributed to variability in fracture resistance measurements and led to relatively high standard deviations. The present study corresponds to the clinical procedure and the natural conditions in the oral cavity in some aspects, since manual polishing similar to the clinical procedure was used instead of mechanical polishing, a load of 10 kg and integrated thermocycling. An artificial periodontium was fitted to the teeth to simulate natural tooth mobility. Its thin, even coating can be used to sufficiently mimic natural tooth mobility [59]. In order to make the results of the chewing simulation and the fracture resistance test comparable, the layer thicknesses of the restorations were designed according to those of earlier investigations with anatomically shaped occlusal surfaces [3, 21]. It should be noted that the manufacturer's specifications are 0.5 mm, which were undercut with 0.3 mm in the fissures to create an anatomy close to that of natural teeth with fissures. Limitations of the study are the artificial antagonists, in the form of steatite ceramic balls, which only had occlusal contact on the supporting cusp and were not able to display clinically present A, B, and C contacts. The absence of oral fluids with their possible influence on adhesive bonding and the lack of intraoral wear factors such as abrasive substances in food, chewing behavior and chewing force or parafunctions are also limitations. The sample size in this study was determined based on previous investigations, which employed a similar study design and accounted for comparable sources of variability. These earlier studies demonstrated acceptable standard deviations and enabled statistical differentiation between the factors evaluated [3, 4, 21, 27, 46, 60].

It can be concluded that using material with higher yttrium content for the occlusal veneers increased their survival rate in the chewing simulator. Even though only the E‐LT group had a significantly higher fracture resistance compared with the E‐HT group, the fracture resistance was raised with the increase in yttrium content. The fracture resistance of the occlusal veneers bonded to different substrates did not differ significantly, but the restorations bonded to enamel showed better results than those bonded to dentin with margins in enamel. All the ceramics used showed very low wear after the simulated 5‐year loading and the wear of the antagonists was as similar to the wear in natural dentition, irrespective of the material used.

## 5 Conclusions

Under the limitations of this laboratory study, it can be concluded that ultrathin (0.3 and 0.6 mm) zirconia occlusal veneers with different yttrium contents showed fracture strengths above the recommended minimum values for posterior restorations of 700 N and were independent of bonding to enamel or dentin with margins in enamel [61]. All zirconia restorations showed wear‐resistant behavior. Clinical studies are needed to quantify the survival of ultra‐thin zirconia veneers in the oral cavity.

## Conflicts of Interest

The authors declare no conflicts of interests.

## Data Availability

The data that support the findings of this study are available from the corresponding author upon reasonable request.
